# Validating the knowledge represented by a self-organizing map with an expert-derived knowledge structure

**DOI:** 10.1186/s12909-024-05352-y

**Published:** 2024-04-16

**Authors:** Andrew James Amos, Kyungmi Lee, Tarun Sen Gupta, Bunmi S. Malau-Aduli

**Affiliations:** 1 College of Medicine & Dentistry, James Cook University, Townsville, Australia; 2 College of Science and Engineering, James Cook University, Cairns, Australia; 3 School of Medicine and Public Health, University of Newcastle, Newcastle, Australia

**Keywords:** Artificial intelligence, Machine learning, Curriculum development, Scientometrics, Medical education, Explainable AI

## Abstract

**Background:**

Professionals are reluctant to make use of machine learning results for tasks like curriculum development if they do not understand how the results were generated and what they mean. Visualizations of peer reviewed medical literature can summarize enormous amounts of information but are difficult to interpret. This article reports the validation of the meaning of a self-organizing map derived from the Medline/PubMed index of peer reviewed medical literature by its capacity to coherently summarize the references of a core psychiatric textbook.

**Methods:**

Reference lists from ten editions of *Kaplan and Sadock's Comprehensive Textbook of Psychiatry* were projected onto a self-organizing map trained on Medical Subject Headings annotating the complete set of peer reviewed medical research articles indexed in the Medline/PubMed database (MedSOM). K-means clustering was applied to references from every edition to examine the ability of the self-organizing map to coherently summarize the knowledge contained within the textbook.

**Results:**

MedSOM coherently clustered references into six psychiatric knowledge domains across ten editions (1967–2017). Clustering occurred at the abstract level of broad psychiatric practice including General/adult psychiatry, Child psychiatry, and Administrative psychiatry.

**Conclusions:**

The uptake of visualizations of published medical literature by medical experts for purposes like curriculum development depends upon validation of the meaning of the visualizations. The current research demonstrates that a self-organizing map (MedSOM) can validate the stability and coherence of the references used to support the knowledge claims of a standard psychiatric textbook, linking the products of machine learning to a widely accepted standard of knowledge.

**Supplementary Information:**

The online version contains supplementary material available at 10.1186/s12909-024-05352-y.

## Background

The selection of content for inclusion in the medical curricula of undergraduate university degrees, as well as the curricula of postgraduate generalist and specialist programs for training doctors, is almost entirely based on expert judgement rather than empirical evidence about the relative importance of different medical skills and knowledge for competence in those areas of practice [1, 2]. This is largely due to the enormous set of potentially relevant information from which the topics covered by medical curricula must be selected, the rapid accumulation of new information across diverse topics, and the partially mutual, partially conflicting interests of stakeholder groups including patients, clinicians, and decision-makers.

An example of the reliance on expert judgement for the selection of content is curriculum mapping, which describes a type of structured brainstorming intended to reduce the chance that important topics will be left out of a curriculum. It advises developers to consider the needs of different stakeholders such as students and teachers, and different curriculum purposes such as learning and assessment [1]. By contrast, while we have not found any research which attempts to do this, it is technically possible to select curriculum content based on empirical evidence, defined as systematically gathered and evaluated evidence about quantitatively or qualitatively observable phenomena. An example for the purposes of illustration is a medical curriculum developed to address only the 100 diagnoses associated with the largest set of costs in a health care system. While it is unlikely that such a curriculum would be acceptable to patients, physicians, or administrators, there may be advantages to a curriculum designed by experts who systematically integrated empirical information such as resource-intensive diagnoses into content selection decisions.

In the absence of the widespread use of empirical information to guide content selection, medical curriculum development largely relies upon expert judgement. There is face validity to having domain experts such as physicians, surgeons, or psychiatrists decide what knowledge and skills are core to their practice. However, basing medical practice on expert judgement alone can have negative consequences, particularly where biases are widely shared. For example, it is now known that the previously common practice of excluding women from patient samples in medical research was based on the erroneous assumption that patterns of health, illness, and response to treatment were common to men and women, leading to widespread sub-optimal treatment of cardiac health and illness in women spanning decades [3].

While there is no doubt that the peer-reviewed literature itself is subject to biases, the provision of objective sources of evidence summarizing features of the research in each field of medicine and their place within medicine more generally could reduce bias in two ways. First, the least biased expert would surely be the one with the best knowledge of an entire field of research. An objective summary of the available research could highlight for the expert areas they have overlooked, including topics of emerging importance, or areas they have overvalued, such as treatments associated with declining research, and help organize their assessment of the relative importance of different areas. At the worst, it could reassure the expert that they currently have an accurate understanding of the totality of the relevant literature.

Second, empirical models of the research literature can be interrogated for bias in ways that expert judgement cannot. For example, now that it is understood that knowledge of cardiovascular health and disease was biased by the exclusion of women, it may be possible to detect similar biases in other areas of medicine by analyzing the proportion of patients from particular demographic or clinical groups across fields of research. In some cases, the under-representation of women in clinical samples would be expected (research into the detection of prostate cancer, for example) while in other cases it would indicate the possibility of bias indicating follow-up (research into the thresholds for follow-up on screening for heart disease, for example).

The enormous and growing volume of information about medicine means that empirical models will often need to be presented in visual form to be understood. The field of Machine learning (ML) provides techniques capable of producing empirically derived visualizations that meaningfully summarize the large volume of information and level of complexity of databases of medical knowledge. Medline is a freely available database maintained by the National Library of Medicine (NLM) which lists and describes almost all articles published in reliable peer-reviewed medical journals, including articles as far back as the nineteenth century [4]. Skupin has been particularly creative in applying map-making techniques to visualizations of the Medline database to highlight structural features such as the relative frequency with which medical concepts co-occur in research papers [5].

Despite their promise, there are significant barriers to the adoption of ML models in medical practice, including for curriculum development. Most importantly, clinicians, educators, and other health professionals resist using ML models if they do not understand how those models work, or what the models mean [6, 7].

### Visualizing medical knowledge with self-organizing maps

Many ML techniques are used to visualize the knowledge contained within published peer-reviewed scientific literature [8]. Self-organizing maps (SOMs), pioneered by Kohonen, are an attractive method that can be applied to structured or unstructured data, can identify novel patterns not anticipated by researchers, and have successfully projected very high dimensional datasets onto 2D maps while retaining many of the topographical features of the original high dimensional space [5, 9]. These features are suited to eliciting, summarizing, and compactly representing the properties of medical knowledge latent in the published literature as an objective source of evidence to guide the selection of content for inclusion in medical curricula. Supplementary Material 1 presents a summary of the technical features of SOMs.

While far from the only source of useful information about the practice of medicine, the corpus comprising the full set of peer reviewed literature indexed by databases such as Medline [4], Web of Science [10], and Scopus [11], appears likely to be the most comprehensive and authoritative compendium of evidence based medical knowledge independent of the experiences and biases of individual clinicians. If this premise is accepted, then maps derived using ML methods to summarize and organize that evidence have the potential to provide the most reliable empirical basis with which to guide the selection of content by experts engaged in curriculum development. To be useful for curriculum development, maps of medical knowledge must provide experts with access to readily understandable information about the structure and characteristics of the medical literature that are not otherwise available, and that are consistent with experts’ current understanding of the literature.

Evaluation of existing efforts to map the medical literature have focused on technical features such as the accuracy of clustering [12, 13] or the visual idioms of presentation [5] more than the meanings conveyed by the maps to experts attempting to integrate them into their own practice. As an example of the latter, if a cardiovascular expert involved in curriculum development became aware that women had been excluded from earlier cardiac research, it would be useful for them to be able to consult a map of the peer-reviewed research about the treatment of heart disease which color-coded the representation of other groups previously under-represented in medical research. Such a map would make the distribution of adequate- versus under-representation of specific groups in specific areas of research intelligible to the expert, who could then draw on their expertise to decide what use to make of that information. The current research is a necessary step towards this type of map.

### Integrating self-organizing maps into the curriculum development process

Thus, SOMs are powerful analytic tools with the potential to facilitate curriculum development by condensing enormous sets of information into 2-dimensional maps which highlight relevant information using visual cues like color. Translating this potential into practice requires several steps. The first step is to show it is technically possible to model the entire Medline database in a single SOM. Amos et al. (2023) used SOMs to visualize the entire corpus of peer-reviewed medical literature indexed by the database Medline [14]. Amos et al. (in preparation) extended their original SOM using the Relative Density SOMs (ReDSOMs) technique developed by Denny et al. (2010) to show how the knowledge indexed by Medline evolved over time [15].

Amos et al. (2022) further extended the approach by identifying psychiatric topics of emerging importance in the Medline database between 1972 and 2016 [16]. Also planned is an experimental test of whether the emerging topics identified by Amos et al. (2022) are perceived to be useful for the purpose of planning professional development sessions by a group of psychiatrists.

The current research describes a novel method of validating the SOM developed by Amos et al. (2023) by examining its ability to meaningfully interpret the knowledge represented by the expert-selected references across the editions of a psychiatric textbook. The ultimate goal is to develop SOMs in forms useful for integration into curriculum development or curriculum maintenance activity, for example by highlighting emerging topics for consideration for inclusion in a curriculum; or by highlighting topics of declining importance for consideration for removal.

### Study context

The ML field is starting to realize that the understandability and acceptability of its models is the main factor in determining whether they will be used by experts [17–19]. Evaluations of current ML models of the medical literature generally focus on technical features such as accuracy rather than indicators of understandability and acceptability such as the meaning of the models to end-users. We developed a novel approach to the validation of a SOM of the medical literature by examining whether it meaningfully organized the implicit knowledge structures represented by reference lists of different editions of a psychiatric textbook.

For the purposes of this research an explicit knowledge structure in a set of articles is any relationship between two or more articles likely to be immediately obvious to a human reader. For example, a human reader will immediately recognize the grouping of articles by the journal in which they are published. An implicit knowledge structure is any pattern of relationships between articles that is unlikely to be readily apparent to a human reader, but is detectable by an ML technique like a SOM. An example of an implicit knowledge structure is a bias against the inclusion of female participants in a particular type of treatment trial that is revealed by the visual properties of a SOM. It should be noted that we are not referring to explicit/implicit knowledge as it exists in a human brain according to cognitive psychological theories, but the structures of knowledge reflected in the weights of a ML algorithm.

### Expert-derived knowledge structure

*Kaplan and Sadock’s Comprehensive Textbook of Psychiatry* is the dominant textbook for the study of psychiatry in the US and elsewhere (afterwards referred to as *KSCTP*) [20]. First published in 1967, the tenth edition arrived in 2017 [21], with new editions appearing somewhat irregularly but generally close to 5 years apart. Usefully for the validation of a map of the medical literature, *KSCTP* has included detailed reference lists in support of its knowledge claims starting with the first edition. We hypothesized that a SOM of medical knowledge trained on the MeSH of the complete set of Medline articles would meaningfully interpret the knowledge structures implicit in the references published within each edition of *KSCTP*. For example, we expected that the complete set of references within each edition of *KSCTP* would be represented by specific regions of the SOM which would change in a coherent and understandable way over time.

### Purpose of the study

This paper aims to demonstrate that SOMs can meaningfully visualize features of psychiatric knowledge contained within the peer reviewed literature consistent with the organization implicit in the reference lists of the independently developed expert derived textbook *KSCTP*. In previous work the same authors applied a SOM of 350 × 350 nodes to the complete set of articles indexed by the Medline database to show the relative importance, relationships between, and evolution of, domains of medical knowledge represented by Medical Subject Headings (MeSH) defined by the NLM (Amos AJ, Sen Gupta T, Lee K, Malau-Aduli B: Mapping the evolution of psychiatric knowledge indexed in Medline 1900–2022 using self-organising maps, in preparation); and used a custom machine-learning algorithm developed by Ohniwa et al. [22] (the incremental statistic) to identify topics of emerging importance in the psychiatric subset of the Medline indexed literature [23].

Conceptually, the SOM represents the knowledge contained across all articles indexed by Medline, while each edition of the *KSCTP* textbook is represented by the references selected as the authority for the knowledge claims by individual authors across a period of fifty years. Before training, the SOM weights represented a random organization of knowledge. After training, the SOM weights represent the organizational structures of the entire database. If the organizational structures learned by the SOM are consistent with the knowledge claims of the textbook authors, the SOM will group similar articles together; while if they are not consistent, the SOM will randomly distribute articles across the 2-d map. The division of the references across all ten editions of the textbook adds another layer of validation by showing how stable the clusters of knowledge structures are over time, independent of the individual references.

The current paper is designed to investigate whether the relationships extracted from the Medline database by SOMs are consistent with the organization of psychiatric knowledge across ten editions of a core psychiatric textbook, answering the research questions:

1. Is a SOM trained to extract the implicit organizational structures of medical research indexed by the Medline database consistent with the implicit expert-derived organizational structures of a core psychiatric textbook?
2. How does the interpretation of the psychiatric knowledge represented by each edition of the textbook provided by its projection onto the SOM model change across those editions?

These research questions can be expressed in the form of the hypotheses:

1. The SOM will cluster conceptually similar articles from the *KSCTP* close together, and conceptually different articles far apart for the total set of references, and for the set of references specific to each edition.
2. The clusters of conceptually similar articles of each edition of the *KSCTP* will overlap between editions in ways that can be interpreted with reference to broad changes in psychiatric knowledge domains.

## Methods

### Mapping the knowledge covered by a psychiatric textbook

The changes between editions of the core psychiatric textbook *Kaplan and Sadock’s Comprehensive Textbook of Psychiatry* were visualized by projecting the articles contained within the reference lists of the entire textbook:

- A complete set of references from all editions of the textbook was projected onto the static SOM from Amos et al. (2023)
- Reference sets from each edition of the textbook were projected onto the SOM

### Training the self-organizing map of the medical literature (MedSOM)

As we have described in more detail elsewhere [14], the SOM for this research comprised a set of nodes arranged in a square 2D matrix (350 × 350 nodes). This shape was chosen during the previous research by experimentation with different sizes, starting with the 275 × 275 nodes of an earlier model which was trained on a smaller subset of the Medline database than ours, and increasing up to 400 × 400 nodes by adding 25 nodes in each dimension. With increasing size, the topographic error of the model first declined and then reached a plateau at 350 × 350 nodes, leading us to select this size of map for the current research.

The training set included the entire set of 33,375,863 peer-reviewed articles published by the NLM in its Medline database as of 1.1.2022. Each article was represented as a binary vector with 29,917 elements encoding the presence or absence of each of the 29,917 Medical Subject Headings (MeSH) in the set published by the NLM as of 1.1.2022, after excluding administrative codes and MeSH annotating less than 100 articles.

The MeSH are a set of phrases with defined meanings maintained as a controlled vocabulary by the NLM. They are organized in a tree-like hierarchy with categories such as “Anatomy,” “Diseases,” and “Disciplines and Occupations” at the top, with increasingly specific categories at lower levels. For example, one of the pathways under “Diseases” narrows its meaning through the phrases “Infections,” “Bacterial infections,” and “Bacterial Zoonoses” at the lowest level. The NLM controls the meaning of each MeSH and their organization into a hierarchy. They continuously review the peer-reviewed medical literature for new phrases to be defined and added to their controlled vocabulary and its hierarchy. One of the top-level categories is “Psychiatry and Psychology” [23].

On average, each of the 33 million articles had been annotated with 9 MeSH describing its main features. For example, an article describing a clinical trial of high-dosage haloperidol for patients with chronic schizophrenia would be annotated with MeSH representing: “Adult population, Human,” “Schizophrenia,” “Haloperidol,” among others. For SOM training purposes, this article would be represented by a vector of 29,917 binary elements in which ~ 29,908 would be false/0 (indicating that the MeSH did not describe the article) and ~ 9 would be true/1 (indicating that the MeSH did describe the article – see Supplementary Material 1).

Kohonen’s batch training algorithm was implemented using sparse matrices [9, 14, 24, 25]. In each epoch, every article was presented to the SOM, which calculated the best matching unit and the second-best matching unit, with error accumulated over all articles and weight changes applied at the end of the epoch, using the algorithm described in Table 1.

Twenty training epochs were completed, with calculation of the topographic error after each epoch. The codebook with the lowest topographic error was selected for further analysis. The nodes comprising the MedSOM represented by this codebook were divided into those representing relatively psychiatric knowledge and relatively non-psychiatric knowledge. Nodes where the input weights gave a higher priority to psychiatric than non-psychiatric MeSH were categorized as psychiatric, with all others categorized as non-psychiatric. Psychiatric MeSH are those organized as sub-categories under the top-level “Psychiatry and Psychology” category in the NLM’s controlled vocabulary hierarchy.

**Table 1:**
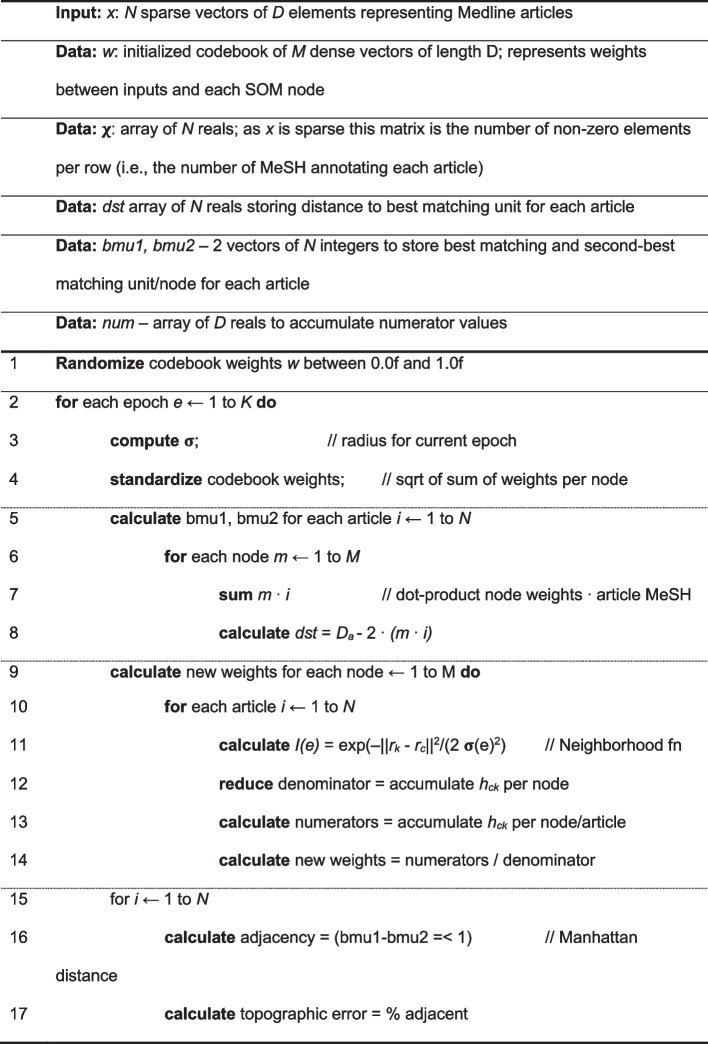
Training algorithm – Parallel Batch SOM with sparse binary matrices

D - total number of MeSH categories (=29,917–; D_a_ - number of MeSH categories annotating an individual article; N - number of articles (=33,375,866); M - number of nodes (350x350=122,500); –(e) - width of the neighborhood (which changes over training epochs according to the formula 175/(1.7)^epoch^)

### Projecting textbook editions onto a published medical literature map

Each edition of the core psychiatric textbook *KSCTP* reports detailed reference lists comprising the scientific evidence base for the asserted knowledge claims. The citations include books, peer reviewed articles, and grey literature including government reports, web pages, and other sources. Complete reference lists were obtained from each of the 10 editions published before 2023, and the R programming package easyPubMed was used to retrieve the unique PubMed ID (PMID) and list of Medical Subject Headings (MeSH) describing each referenced article. Note that while PubMed and Medline are often used interchangeably, technically Medline is the database and PubMed is the online vehicle for searching Medline. We will use Medline hereafter except where PubMed is part of a technical name or programming instruction.

Table 2 describes the structural features of the *KSCTP *textbook [21]. It maintained a standard edited format across all editions, with a low of 50 chapters in 1989 (5th edition) and a high of 62 chapters in 2017 (10th edition). Single or multiple invited authors were responsible for subsections of each chapter, and the number of subsections per chapter varied from a low of 4.39 in 1967 (1st edition) to a high of 6.85 in 2005 (8th edition). There was an average of 7.3 new chapters added to each edition, with an average of 6.3 chapters either removed or merged, with more changes in earlier editions. On average, 26% of PubMed citations persisted from one edition to the next, with a low of 5% in 1985 (4th edition) and a high of 55% in 1980 (3rd edition).

**Table 2:** Organizational features of *Kaplan & Sadock*^*21*^ textbook across editions

Edition	Year	Pages	Chapters	New Chapters	Removed chapters	Citations	Citations per chapter	Medline citations	% Persisting citations
1st	1967	1629	53	53	N/A	2927	55.2	585	N/A
2nd	1975	2572	52	18	19	9126	175.5	2280	44%
3rd	1980	3306	57	9	4	17,030	298.8	4225	56%
4th	1985	2055	54	3	6	2091	38.7	407	5%
5th	1989	2158	50	10	14	3999	80.0	780	21%
6th	1995	2805	53	8	5	8625	162.7	2393	16%
7th	2000	3345	55	7	5	10,180	185.1	4257	23%
8th	2005	4064	55	2	2	11,795	214.5	3780	20%
9th	2009	4521	59	4	0	10,472	177.5	4815	25%
10th	2017	4533	62	5	2	9235	149.0	4157	26%

The complete text of each reference was submitted to the Medline search function using the get_pubmed_ids function within the r package *easyPubMed*, and the complete xml of each match was retrieved using the easyPubMed::fetch_pubmed_data function. The accuracy of retrieval was confirmed by manually checking that the first author, title, and journal specified in the reference text matched those fields in the retrieved information. Non-matching references were discarded, and the number of references in each edition confirmed/not confirmed to be indexed by the Medline database were recorded. As only references which were matched in the Medline database were retained, only Medline-indexed articles were included in the analysis. Records were kept of both the number of Medline-indexed references and the total number of references for each edition.

### Interpreting the meaning of the projection of textbook knowledge onto MedSOM

MedSOM is a 2-dimensional representation of the organization of all the information contained within the Medline database, comprising 350 × 350 = 122,500 nodes laid out in a square grid (see Supplementary Material 1 for a description of the technical features of SOMs). Each of the individual nodes is characterized by a set of 29,917 weights which each represent one of the 29,917 MeSH used by the NLM to annotate articles. Each article was represented by a vector of 29,917 Boolean variables corresponding to the node weights and MeSH. For each Boolean, a true value indicated that an article was annotated with a specific MeSH, while a false value indicated the article was not annotated with that MeSH. Just as each article was annotated with on average 9 MeSH, so the representation of the article in the SOM was a Boolean vector with on average 9 true and 29,908 false, Boolean variables. After training, nodes close to each other in the SOM had similar MeSH weights, and therefore were more likely to be the best matching unit for articles close together in the high-dimensional space represented by the annotating MeSH [9].

For the purposes of providing an external validation of the organizational structure inferred by the SOM it is necessary to show that it can coherently organize the knowledge structures implicit in some other set of information. In the current study, that set of information is the complete set of articles referenced across all editions of the *KSCTP* textbook, and within each edition.

The list of MeSH describing each retrieved article was input to the MedSOM to identify the node best representing that article. The structure of knowledge represented by these articles was then projected onto MedSOM as a single set containing all editions, and individually for each edition. K-means clustering was applied to the position of each article on the MedSOM grid for the complete set and individual edition-sets of articles to identify groups of related articles, using the *kmeans* function in the r *stats* package.

For each of the complete set and individual edition-sets of articles the optimal number of clusters was determined using the r *stats* package’s *fviz_nbclust* function using the “elbow” method. The elbow method seeks to minimize both the number of clusters and the sum of squares of the distance between articles within each cluster by finding the cluster number beyond which there is a relative plateau in further reductions of sums of squares.

The success or failure of the SOM to provide a meaningful interpretation of the expert-derived organizational structures of the *KSCTP* textbook can be judged by the coherence of the interpretation provided for the full set of references across all editions, and its persistence between editions. Before training, the SOM would be expected to distribute articles at random across its nodes. After successful training, it would cluster articles covering similar topics close together, both within each edition, and across all editions.

The meaning of the knowledge clusters was explored by extracting the most common MeSH annotating articles in each cluster and identifying the 10 articles in the cluster with the largest number of those common MeSH.

## Results

**Fig. 1:**
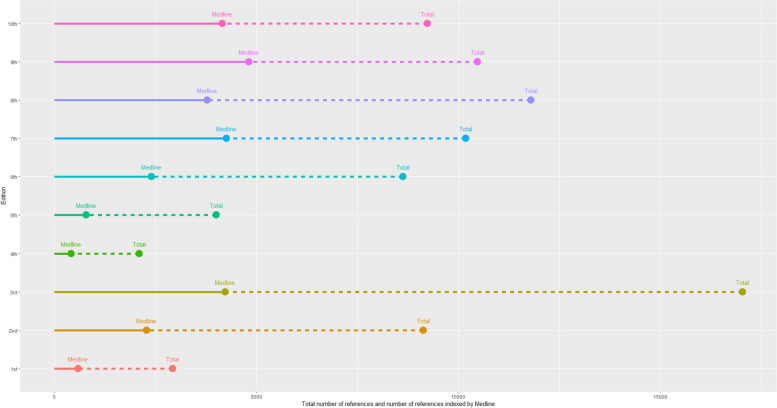
Number of references by edition (Medline indexed references and Total references indexed by edition)

Figure 1 shows the total number of references in the reference lists of each edition of *KSCTP*, alongside the number of those references indexed by the Medline database. As the Medline database only indexes scientific articles published in peer-reviewed medical journals, the difference between total and Medline indexed references in each edition is made up of non-indexed citations such as books, newspaper articles, and grey literature.

The discontinuity after the third edition with a large reduction in the total and indexed references was due to a conscious decision by the editors of the *KSCTP*textbook. The rationale provided in the fourth edition foreword was that the rapidly escalating number of references over the first three editions had achieved the desired result of establishing the authoritative nature of the textbook grounded in the published literature, allowing for more selective citations in future editions [26]. From the very low baseline of the fourth edition there was a steady increase in the number of total references and references indexed in Medline until the 8th edition, after which numbers stabilized at close to 10,000 total references and close to 5,000 Medline indexed references. Thus, in later editions of *KSCTP*, about half of the references are indexed, peer-reviewed articles, a significant change from the 3rd edition (published 1980) where less than one quarter of the references were indexed, peer-reviewed articles, with a much higher reliance upon books.

### Consistency, coherence and meaning of SOM projections

**Fig. 2:**
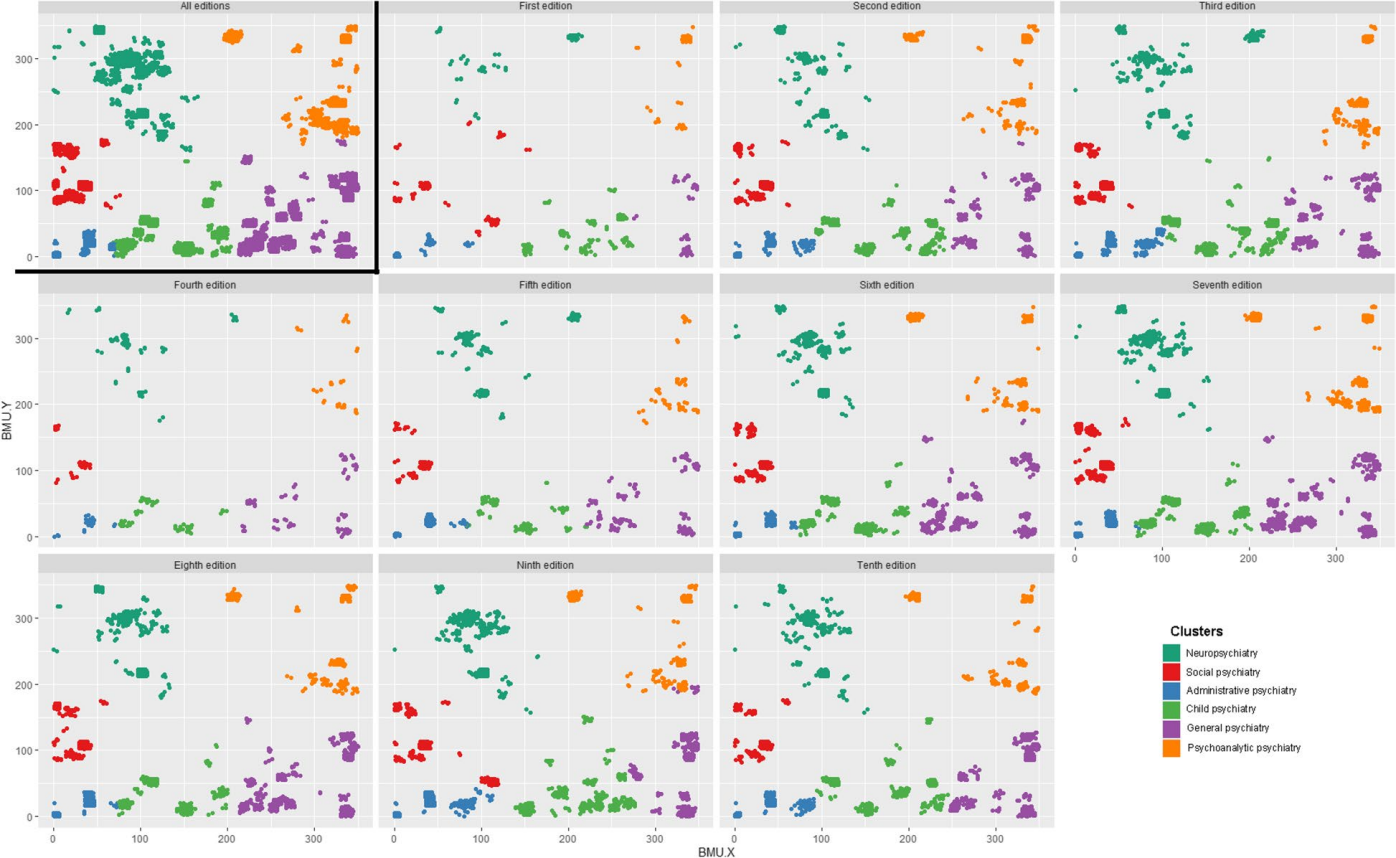
Knowledge clusters by edition

**Fig. 3:**
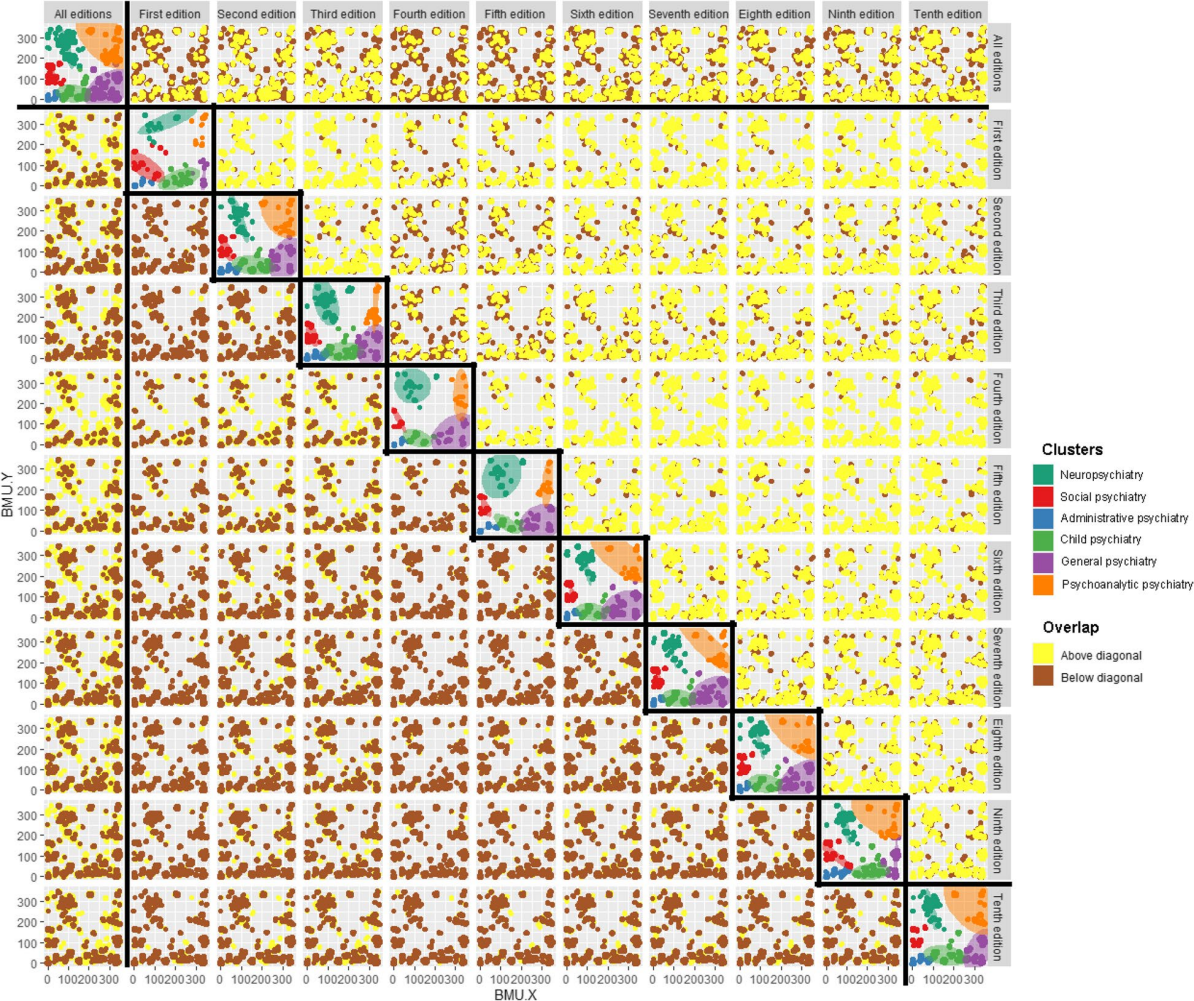
Knowledge cluster overlap—overall and by edition

The purpose of this research is to show that the SOM trained on all Medline articles and MeSH provides a meaningful, coherent, and consistent interpretation of the knowledge contained within the *KSCTP*. In this exploratory phase, coherence is demonstrated by an understandable organization of the knowledge into identifiable categories; consistency is demonstrated by the persistence of the same identifiable categories over different editions of the *KSCTP*; and meaning is inferred by examination of the qualities of the articles clustered together on MedSOM. Coherence and consistency are demonstrated in Figs. 2 and 3, while meaning is inferred from Tables 3 and 4. While future phases of research will focus on optimizing formal properties of the SOM, that is not the goal of this phase of research.

**Table 3:** Cluster-defining medical subject headings

Cluster MeSH /Edition	Neuropsychiatry	Social psychiatry	Administrative psychiatry	Child psychiatry	General/adult psychiatry	Psychoanalytic psychotherapy
1	Oxygen Radioisotopes	Beneficence	False Positive Reactions	Appetite Regulation	Adult	**Depressive Disorder**
2	Regional Blood Flow	Ethical Theory	False Negative Reactions	Behavior	Mental Disorders	Dopamine
3	Tomography, Emission-Computed	History, 20th Century	Psychiatric Status Rating Scales	Child	Middle Aged	Latency Period, Psychological
4	Behavior, Addictive	Personal Autonomy	Mental Disorders	Child, Preschool	Shame	Psychosexual Development
5	Circadian Rhythm	Physician–Patient Relations	Diagnosis, Differential	Conditioning, Operant	Hospitalization	Ego
6	Cues	Behavior Therapy	Psychometrics	**Enuresis**	Psychiatric Status Rating Scales	Electroconvulsive Therapy
7	Memory	**Bipolar Disorder**	**Schizophrenia**	Food	Attitude to Health	Norepinephrine
8	Nervous System Physiological Phenomena	Continuity of Patient Care	Sensitivity and Specificity	Infant	**Borderline Personality Disorder**	Psychotherapy, Group
9	Neuropsychological Tests	Ethical Analysis	Antipsychotic Agents	Migraine Disorders	Diagnosis, Differential	**Depression**
10	**Alzheimer Disease**	Euthanasia, Active	**Borderline Personality Disorder**	Weight Loss	Guilt	Individuation

*Medical Subject Headings (MeSH) associated with a psychiatric diagnosis indicated in Bold*

**Table 4:** Best matching articles by cluster

Cluster	Article titles
Neuropsychiatry	A biologist examines the mind and behavior **Psychosurgery today: psychiatric aspects** Serotonin, cerebral blood flow, and cerebral metabolic rate in geriatric major depression and normal aging **Changes in regional cerebral blood flow elicited by craving memories in abstinent opiate-dependent subjects** Frontal lobotomy in early schizophrenia. Long follow-up in 415 cases **The dementia of dementia praecox** Effect of schizophrenia on frontotemporal activity during word encoding and recognition: a PET cerebral blood flow study **Sertraline. A review of its pharmacodynamic and pharmacokinetic properties, and therapeutic potential in depression and obsessive–compulsive disorder** Limbic activation during cue-induced cocaine craving **Mechanisms of lithium action** An evaluation of bimedial leucotomy **Estrogen-serotonin interactions: implications for affective regulation** Initial masking of organic brain changes by psychic symptoms: clinical and electroencephalographic studies **A long-term follow-up of schizophrenics treated with regressive ECT** Transduction of psychosocial stress into the neurobiology of recurrent affective disorder **Dopamine receptor binding predicts clinical and pharmacological potencies of antischizophrenic drugs**
Social psychiatry	The ethics of care and treatment of sex offenders **Three types of peer tutoring: effects on the attitudes of students with learning disabilities and their regular class peers** Comorbidity of personality disorders and depression: implications for treatment **Longitudinal patterns of anxiety from childhood to adulthood: the Great Smoky Mountains Study** Empathy: misconceptions and misuses in psychotherapy **The psychotherapeutic utility of the five-factor model of personality: a clinician's experience** Escalation of aggression: experimental studies **Substance abuse and adolescent suicidal behavior** Demonstrating translational research for mental health services: an example from stigma research **An improved detoxification technique for heroin addicts** Dealing with our losses **Group therapies for nursing home adults: an evaluation of two treatment approaches** Empirically supported treatments for children with phobic and anxiety disorders: current status **Increased depressive ratings in patients with hepatitis C receiving interferon-alpha-based immunotherapy are related to interferon-alpha-induced changes in the serotonergic system** Cognitive-behavioral treatment of school phobia **Preliminary report on the application of contingent reinforcement procedures (token economy) on a "chronic" psychiatric ward** AMERICAN PSYCHIATRY AND THE CRIMINAL: A HISTORICAL REVIEW **The reinforcement of behavior in institutional settings** The effects of social skills training and peer involvement on the social adjustment of preadolescents **The consequences of open and closed adoption for older children** Clinical considerations in group treatment of narcissistic disorders **Behavior therapy and sex therapy**
Administrative psychiatry	Citizen participation in the development of a community mental health center **The right to refuse treatment with antipsychotic medications: retrospect and prospect** Field trial for autistic disorder in DSM-IV **Gaps in doctor-patient communication. Patients' response to medical advice** The physician-elderly patient-companion triad in the medical encounter: the development of a conceptual framework and research agenda **Discussion of medical errors in morbidity and mortality conferences** Performance of screening and diagnostic tests. Application of receiver operating characteristic analysis **Treating substance-use disorders among physicians** Limitations of listing specific medical interventions in advance directives **On wearing two hats: role conflict in serving as both psychotherapist and expert witness** The ethics of therapeutic modality choice **Routine laboratory testing for medical disorders in psychiatric inpatients** Pharmaceutical care role model in psychiatry–pharmacist prescribing **Application of the predictive value model in the analysis of test effectiveness** Death due to treatment **Predictive validity of certification by the American Board of Internal Medicine**
Child psychiatry	Ontogenetic development of the human sleep-dream cycle **Incontinence and enuresis** Comorbidity of parental anxiety disorders as risk for childhood-onset anxiety in inhibited children **The effectiveness of group psychotherapy with children** Short-term group psychotherapy for children: an overview **Anxiety sensitivity and panic disorder** Tests of competency to consent to treatment **Developmental dyscalculia: a brief report on four cases** The borderline diagnosis in adolescents: symptoms and developmental history **The adolescent and the "hidden" parent** A follow-up report on children who had atypical sexual experience **A general test of motor impairment for children** Conditions for versatile learning, Helmholtz's unconscious inference, and the task of perception
Adult Psychiatry	Schizophrenia in the National Academy of Sciences-National Research Council Twin Registry: a 16-year update **Psychosis as a state of aberrant salience: a framework linking biology, phenomenology, and pharmacology in schizophrenia** Patient refusal of hydration and nutrition. An alternative to physician-assisted suicide or voluntary active euthanasia **Alcohol and temporal lobe dysfunction. Some of its psychomotor equivalents** Defeminization and adult psychological well-being among male homosexuals **Commonly prescribed medications and potential false-positive urine drug screens** Shame and humiliation in the medical encounter **Detection, prevention and retardation of menopausal osteoporosis** Unmet service needs in methadone maintenance **Overview: the "wife-beater's wife" reconsidered** Morbidity following sudden and unexpected bereavement **The value of psychiatric treatment: its efficacy in severe mental disorders** DEPRESSION AMONG MEDICALLY ILL PATIENTS **The irritable bowel syndrome. A clinical review and ethical considerations** Predictors of posttraumatic stress disorder and symptoms in adults: a meta-analysis **THERAPEUTIC EFFICACY OF ANTIDEPRESSANT DRUGS. A REVIEW** Psychoendocrine research on sexual orientation. Current status and future options **Efficacy of combinations of intramuscular antipsychotics and sedative-hypnotics for control of psychotic agitation** Neuroleptic-associated tardive syndromes **Contemporary conversion reactions: a clinical study** Psychiatric observations under severe chronic stress
Psychoanalytic psychiatry	Comorbidity of personality disorders and depression: implications for treatment **A biologist examines the mind and behavior** Etiological factors in female transsexualism: a first approximation **Social influences on "self-stimulatory" behavior: analysis and treatment application** The psychotherapeutic utility of the five-factor model of personality: a clinician's experience **How effective are interventions with caregivers? An updated meta-analysis** Personality disorders and treatment outcome in the NIMH Treatment of Depression Collaborative Research Program **Thumb and finger sucking** A self-control behavior therapy program for depression **A verbal group technique for ego-disturbed children: action to words** Behavior therapy and sex therapy **Child care and attachment: a new frontier the second time around** Activity-interview group psychotherapy: theory, principles, and practice **Psychotherapy of borderline psychotic children** A study of Fairbairn's theory of schizoid reactions **Contemporary conversion reactions: a clinical study** Group therapy for schizophrenia: a practical approach **Clinical considerations in group treatment of narcissistic disorders** A developmental-genetic analysis of common fears from early adolescence to early adulthood

Figure 2 shows the results of the projection of all articles referenced by *KSCTP* onto the MedSOM, then subject to k-means cluster analyses, where each separate cluster is color-coded. The first facet shows the projection and clustering of all references from all editions, and the other facets each show the results using references from a single edition. The six clusters describe coherent and separate domains of psychiatric knowledge which are stable across the editions. The MedSOM does show variations consistent with the underlying data set, for example by a rapid increase in the density of articles across the first three editions, and then a steady increase from the fourth to eighth editions followed by a plateau.

Figure 3 reproduces the facets of Fig. 2 along the diagonal cells and shows the superimposition of all the articles in each edition on all the articles of each of the other editions. The smaller size of each cell of the matrix in Fig. 3 made the points in the scatter plots of Fig. 2 difficult to compare across editions. The *stat_ellipse* function of the R *ggplot2* package was used to superimpose a shaded normal ellipse containing 95% of the articles in each domain using the same color coding. There is a high degree of overlap between the position of articles across editions. To show where there is overlap and where there are differences between editions, the cells beneath the diagonal show the editions labelled on the y-axis in yellow above the editions labelled in brown on the x-axis. The cells above the diagonal reverse this pattern.

Beneath the diagonal the first column shows where each individual edition overlaps the complete set. As expected, the first edition does not cover much of the content of the complete set of information, but the second and third editions rapidly increase coverage. While the third edition has the greatest number of references and therefore covers most of the points of the complete set, it least well covers the Neuropsychiatry and Psychoanalytic Psychiatry sections. Conversely, the tenth edition covers all clusters relatively well while also containing areas of non-overlap across all clusters.

The third edition pattern is the result of changes in the knowledge base and practice of psychiatry around 1980, the year of publication of the third edition, as well as the third edition of the Diagnostic and Statistical Manual of Mental Disorders, which radically changed the nosological approach of mainstream psychiatry [27]. The third edition pattern reflects an increasing focus on the basic sciences underlying mental disease and a decreasing focus on psychoanalytic psychotherapy. The tenth edition pattern is consistent with the stabilization of the psychiatric knowledge base with the non-overlapping sections representing all the areas of knowledge and clinical practice which have fallen out of use since 1967.

Another factor likely to have influenced this pattern is the rapid increase in publication rates over the decades since 1967. The NLM reports that Medline registered 186,843 citations in 1967 and 986,012 citations in 2021 [28]. As the MedSOM was trained on all articles indexed by Medline, the structures of knowledge present in 1967 (when the 1st edition of *KSCTP* was published) may have less impact on the MedSOM than structures in 2017 (when the 10th edition was published).

Table 3 reports the 10 most common MeSH characterizing each of the 6 clusters found for each individual edition. Table 4 collects articles with the largest number of common elements for each cluster across all editions. These tables describe the meaning of the organization of knowledge extracted from the Medline database by the MedSOM. For example, articles in the top left Neuropsychiatry cluster are labelled with MeSH related to neuroscience, particularly neuroimaging terms such as Oxygen Radioisotopes and Regional Blood Flow, while articles in the lower-left Social Psychiatry cluster are labelled with social scientific MeSH such as Beneficence and Ethical Theory.

A notable feature of the pattern of MeSH most characteristic of each cluster is the relative absence of diagnostic labels. While Table 3 does include a small number of diagnoses, such as Bipolar Disorder in the Social Psychiatry and Schizophrenia/Borderline Personality Disorder in the Administrative Psychiatry cluster, they appear to be grouped based on features other than their clinical manifestations. For example, the inclusion of Schizophrenia and Borderline Personality Disorder in the Administrative Psychiatry cluster can be explained as the result of their importance in epidemiological studies focused on differential diagnosis rather than symptoms or treatment.

## Discussion

The relatively opaque information provided by many ML visualizations of scientific knowledge suggests that the validity of maps of scientific knowledge depends to a large degree on how easily users can perceive the meaningful patterns they summarize [29]. The current research demonstrates that the semantic structures implicit within the Medline database of peer-reviewed medical literature and made explicit by the MedSOM coherently organize the peer reviewed articles cited as references in a core psychiatric textbook. The organizing structure is consistent across all editions and within individual editions, with variations over time that align with actual changes in the clinical and scientific framework underpinning psychiatric practice in the decades between 1967 and 2017.

Comparing the projection of references from different editions of *KSCTP* shows that MedSOM effectively clusters groups of articles with similar content close together, and those with less similar content further apart. Considering the most common MeSH and actual articles clustered together on the MedSOM provides an understanding of the meaning of the underlying 2D distribution of the map. For example, the growth of the Neuropsychiatry domain and the decline of the Psychoanalytic Psychotherapy paradigm is reflected by changes in the extent of overlap of those clusters leading up to the pivotal year 1980.

One of the more useful features of the SOM approach to mapping of the medical literature revealed by our findings is that it identifies previously implicit structures of knowledge linking sets of articles while simultaneously identifying the categories by which they are linked. For example, the articles grouped close together in the Administrative Psychiatry cluster cover a very broad range of clinical and service situations. The associated MeSH are dominated by technical concepts such as False positive/False negative reactions (the absence/presence of a condition when a test falsely indicates that the condition is present/absent) and Psychiatric Status Rating Scale indicate that these articles are grouped because of their involvement in extra-clinical research such as epidemiology/nosology, service development, and testing.

The reason it is useful to identify previously implicit structures of knowledge in this way is that it then allows for the further refinement of understanding by superimposing other, independent structures. Supplementary Material 2 illustrates how the superimposition of the projection of the *KSCTP* textbook and a density map of articles likely to report research into gender differences can help identify and reduce gender biases in medical curricula. Having identified regions of the map on which to focus, it will automatically select articles likely to be relevant to this effort.

MedSOM echoes the organizational structure of the *KSCTP* textbook, albeit at a much higher level of abstraction. MedSOM has a "Neuropsychiatry" cluster, similar to the "Neural Sciences" section which opens all editions of the textbook. There are clusters specific to "Child psychiatry", and to "General/adult psychiatry". Psychiatry of old age is not separately represented and appears to be subsumed partly within the "General/adult psychiatry" cluster and partly within the "Neuropsychiatry" cluster. The "Social psychiatry" and "Psychoanalytic psychotherapy" clusters match up with chapters on "Contributions of the social sciences" and "Contributions of the psychological sciences". The "Administrative psychiatry" cluster incorporates elements from multiple chapters on the more systemic and technical features of psychiatry including "Quantitative and Experimental Methods in Psychiatry", "Theories of personality and psychopathology", "Diagnosis and psychiatry", "Classification in psychiatry", and "Public psychiatry".

The major structural difference between MedSOM and the textbook is that MedSOM does not appear to include any structural information related to diagnostic categories at either the individual diagnostic level or at the level of syndromes. For example, MedSOM does not include discrete sections for psychotic illnesses (such as schizophrenia) versus affective illness (such as depression and anxiety). The textbook relies heavily on diagnostic categories for its chapter structure. This difference appears to be partly due to the relatively small subset of information contained within the textbook reference sets compared with the MedSOM set (~ 20,000 textbook references versus 33 million used for training the MedSOM, including ~ 4 million addressing psychiatric topics) [23]. Much of the knowledge related to diagnostic systems and differential diagnoses is contained within books such as the Diagnostic and Statistical Manual of Mental Disorders [30] which are not indexed by Medline. The focus of textbook references generally concerns cutting edge treatments and review articles which do not differentiate between individual diagnoses.

### Future research – optimizing the model by considering time and entropy

MedSOM is the first iteration of an approach to mapping the entire domain of peer-reviewed medical literature to make currently implicit knowledge explicit and observable by visual cues in two-dimensional maps. While it is encouraging that this form of the model provides a coherent and consistent interpretation of the knowledge contained within the *KSCTP* textbook, the model will need to be refined before it is ready to be integrated into the curriculum development process. In our opinion the two potential refinements most likely to improve the utility of the model are the addition of the capacity to consider the effects of time on the map of knowledge; and the introduction of a formal measure of the extent to which the SOM provides a coherent and consistent organizing structure for medical knowledge, such as entropy.

To understand how a temporal dimension may improve the MedSOM model, consider that one reason the MedSOM's structure does not include individual diagnoses as structural features may be the rapid changes in psychiatric nosology over the 50 years covered by the 10 editions of the textbook. In that time there have been 4 editions of the Diagnostic and Statistical Manual of Mental Disorders with multiple textual revisions, and with major methodological changes, particularly with the 3rd edition, which imposed a much greater focus on reliable diagnosis based on a phenomenological approach [31]; and the 5th edition, which moved away from categorical diagnoses towards a spectrum approach [30]. The MeSH used to identify diagnostic categories over this period have shown similarly rapid changes, which may have affected the ability of the MedSOM to extract stable patterns of knowledge, particularly given that the MedSOM was trained on data including articles dating back to the nineteenth century.

Nevertheless, the current research successfully demonstrates that a SOM trained on the 33 million articles indexed in the Medline database reveals implicit relationships between the subset of articles referenced by a core psychiatric textbook at a high level of abstraction. As the lack of temporal information in the MedSOM appears to have prevented a finer level of detail, extending the current approach to a temporal SOM is a logical next step. The relative density SOM (ReDSOM) approach of Denny et al. develops a longitudinal approach in which a series of SOM are trained using subsets of data divided into time periods, where the trained SOM of one period is used as the initial state of the SOM for the next period [15].

The advantage of this approach is that where knowledge structures persist over consecutive time periods, they will continue to be reflected in the input data and therefore will persist in the SOM. Where knowledge structures change over time, the SOM will learn to replace the old structures with the new. For example, where a diagnosis has changed between time periods, reflected in a new MeSH, the ReDSOM can learn to associate the new MeSH with the existing clinical structures. We intend to extend the current approach to a temporal SOM using the ReDSOM approach. The ability to identify temporal changes in the organizational structure and relationships of the medical and psychiatric knowledge base would provide an empirical trigger for considering the introduction/removal of content into/out of medical and psychiatric curricula based on increasing/declining importance in the literature.

One of the most useful formal properties of network models like SOMs is their entropy, which is a measure of the disorder in a system. The MedSOM before training would be expected to have high entropy and be highly disordered, because each article would be expected to be equally likely to match any individual node. After training, articles with particular MeSH would be expected to be more likely to match nodes in particular regions, increasing the order and decreasing the entropy of the system [32]. Rousseau et al. (2019) discuss how the introduction of entropy to network models can be used to describe the integration of knowledge from multiple disciplines in the novel information represented by individual articles and regions of a SOM [32].

### Limitations

The MedSOM reported in this research extracts relationships from data in an unsupervised manner, so that the meaning of its results have to be inferred. The current approach attempts to provide an external validation of the meaning of the MedSOM with reference to a pre-existing knowledge structure created by experts. While this can strengthen confidence that the MedSOM has imputed structural relationships from the entire set of Medline articles that also apply to the subset of articles referenced by *KSCTP*, it is not directly verifiable. This involves a number of potential limitations. As with the current research, the meaning of the MedSOM may be concentrated at a very high level of abstraction. As a single step in a process of gradually more refined understanding, this is not necessarily a severe limitation, and in the current case, the addition of a temporal dimension has been suggested as a promising way to investigate more detailed levels of meaning in MedSOM and *KSCTP*.

## Conclusions

While maps of scientific and medical literature have great promise as empirical bases for endeavors such as medical curriculum development, their use is constrained by their limited intelligibility to domain experts. The current research demonstrates that an unsupervised SOM derived from the 33 million articles indexed by the Medline database can extract an organizational structure that coherently organizes the knowledge contained within a core psychiatric textbook, at a high level of abstraction. A key limitation of the current research is the use of a SOM trained on a set of articles published over a period of more than one hundred years. The extension of the current approach using a temporal SOM such as Denny's ReDSOM may allow for a more detailed organization of the knowledge indexed by the Medline database that more closely models the expert-defined structures of the *KSCTP* textbook.

### Supplementary Information

**Supplementary Material 1. **

**Supplementary Material 2. **

## Data Availability

All data and materials available on request*.* All analyses undertaken entirely on data freely available at: https://www.nlm.nih.gov/databases/download/pubmed_medline.html.
