# Supplementary material for: Validating the knowledge represented by a self-organizing map with an expert-derived knowledge structure
Source: BMC Med Educ. 2024 Apr 16;24:416. doi: 10.1186/s12909-024-05352-y (PMC11020414; doi:10.1186/s12909-024-05352-y)
Supplement: Supplementary file 1 — Supplementary Material 1. [file 12909_2024_5352_MOESM1_ESM.docx]

Supplementary Material 1: Technical features of Self-organizing maps

| Self-organizing maps (SOMs) are a type of neural network composed of a grid of nodes each of which is an independent unit that processes inputs from the environment and which learn over time to recognize specific features of those inputs. To understand SOMs it is necessary to understand: 1) what a SOM does; 2) the representation of information in the SOM; 3) how individual nodes process inputs from the environment; 4) how the whole SOM learns from inputs; and 5) how the SOM represents what it has learned.  *Supplementary Figure 1: Self-organizing map (8x8 node grid; 4-dimensional input layer; 4 weights per node):*  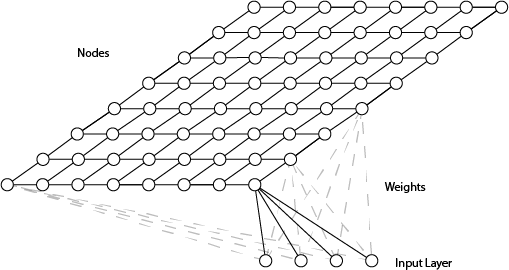  What a SOM does and why this is useful   - A SOM transforms high-dimensional information into a 2-dimensional map which retains many of the properties of the high-dimensional form; In Supplementary Figure 1 the four dimensions of the input layer are transformed into the 2 dimensions of the grid of nodes - In particular, a SOM will retain the property that inputs that were close together in the high-dimensional space will be close together in the 2-dimensional space (topological ordering) - This is useful because human vision is highly effective and efficient at understanding spatial relationships in 2-dimensions and 3-dimensions, but completely unable to understand higher-dimensional relationships   How information is represented in self-organizing maps   - SOMs represent information as vectors, which are ordered sets of numbers of particular size where the position has meaning; in Supplementary Figure 1 the input layer is depicted as a vector of four numbers, and each node receives four weighted inputs from the input layer - The vectors in this research project are sets of 29,917 numbers where the position represents a specific Medical Subject Heading (MeSH) - In computer memory, this looks like a set of 1s and 0s: [0, 1, 0, 0…..(x29,917)] - A vector with a 1/0 in a particular position means the presence/absence of a particular MeSH, for example the vector in the above line might mean: [“Schizophrenia” absent/0, “Bipolar disorder” present/1, “ADHD” absent/0, “Anxiety” absent/0, ….etc]   *Representation of articles:* The representation of an article is the pattern of 1s and 0s in a vector representing all the MeSH annotating that article (as each article is annotated by an average of 9 MeSH, most of the positions will be 0s)  *Vector representing an article (7 numbers shown, 29,910 numbers represented by …):*   \| 0 \| 1 \| 0 \| 0 \| 0 \| … \| 1 \| 0 \| \| --- \| --- \| --- \| --- \| --- \| --- \| --- \| --- \|   *Representation of nodes:* Each node represents a neuron in the brain, which receives information, processes the information, and produces a level of activation. Each node is specified as a vector of the same length as the inputs (29,917 in this research) where each position in the vector represents a weight which is used to process inputs. Weights are not 0s and 1s, but floating-point numbers which vary between certain limits (e.g., between 0.0 and 1.0)  *Vector representing a node (7 numbers shown, 29,910 numbers represented by …)*:   \| 0.11 \| 0.76 \| 0.01 \| 0.003 \| 0.01 \| … \| 0.98 \| 0.001 \| \| --- \| --- \| --- \| --- \| --- \| --- \| --- \| --- \|   How individual nodes process inputs from the environment   - In this research, the inputs are articles represented by vectors which specify the MeSH which annotate those articles (as above) - When an article is presented to the SOM, every node independently processes the information from the vector representing that article - Each node calculates the distance squared between the weight of the node at a particular position, and the article value at that position   Article   \| 1 \| 0 \| 0 \| 1 \| 0 \| … \| 1 \| 0 \| \| --- \| --- \| --- \| --- \| --- \| --- \| --- \| --- \|   Vector -   \| 0.11 \| 0.76 \| 0.01 \| 0.003 \| 0.01 \| … \| 0.98 \| 0.001 \| \| --- \| --- \| --- \| --- \| --- \| --- \| --- \| --- \|   Distance =   \| 0.89 x 0.89 \| (-0.24) x (-0.24) \| (-0.01) x (-0.01) \| 0.997 x 0.997 \| (-0.01) x (-0.01) \| … \| 0.02 x 0.02 \| (-0.001) x (-0.001) \| \| --- \| --- \| --- \| --- \| --- \| --- \| --- \| --- \|   SUM SQUARES = 1.84 [ignoring the other 29,910 MeSH positions]   - The node with the lowest sum of squares after input from a particular article is described as the best-matching unit or BMU; this node is the one where the weight vector is most like the article vector   How the whole SOM learns from inputs   - In the simplest case, before training starts the weights of every node in a SOM will be set to random numbers - As a result, the BMU of each article will be more or less randomly distributed across the grid of nodes, because all article vectors will be equally likely to be similar to all node vectors - The SOM learns by changing the node weights of the BMU after each presentation of an article by moving those weights closer to the article vector; it also moves the weights of the nodes immediately surrounding the BMU towards the article vector, but moves them less than the weights of the BMU - Over multiple iterations of presentation of all articles to the SOM, this process leads to topological ordering of the SOM grid such that inputs that were close together in the high-dimensional space will be close together in the 2-dimensional space (topological ordering; Supplementary Figure 2)   *Supplementary Figure 2: Learning in Self-organizing map:*  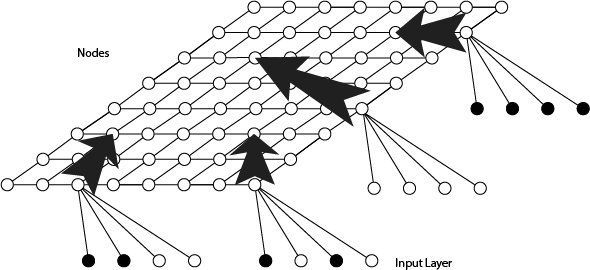  Supplementary Figure 2 shows four inputs with different vectors learning to differentiate themselves over multiple iterations by activating spatially diverse nodes separated on the face of the grid. Black dots represent true/1 and white dots represent false/0 in the inputs. Over learning the BMU shifts from the original random node to a node which reproduces closeness in the high-dimensional space represented by the input (4-dimensions in Supplementary Figure 2) by closeness in the 2-dimensional grid of the SOM. |
| --- | --- | --- | --- | --- | --- | --- | --- | --- | --- | --- | --- | --- | --- | --- | --- | --- | --- | --- | --- | --- | --- | --- | --- | --- | --- | --- | --- | --- | --- | --- | --- | --- | --- | --- | --- | --- | --- | --- | --- | --- |
