# Supplementary material for: Validating the knowledge represented by a self-organizing map with an expert-derived knowledge structure
Source: BMC Med Educ. 2024 Apr 16;24:416. doi: 10.1186/s12909-024-05352-y (PMC11020414; doi:10.1186/s12909-024-05352-y)
Supplement: Supplementary file 2 — Supplementary Material 2. [file 12909_2024_5352_MOESM2_ESM.docx]

Supplementary Material 2 – Identifying implicit bias using self-organizing maps

| One of the most powerful features of SOMs is their capacity to superimpose visualizations derived from different dimensions of the high-dimensional conceptual space on which they are trained. This can be used as a form of high-dimensional Venn diagram that allows for the isolation of areas of knowledge that share specific characteristics in ways useful for curriculum development, for example by identifying areas of research that are both primarily psychiatric, and include significant differences between the sexes.  To illustrate how this could be used for the purposes of reducing the sorts of medical research biases regarding gender noted in the main text, it is useful to consider the existing approach of Sex and Gender Specific Health (SGSH), which “aims to understand sex- and gender-based differences in diseases common to both women and men, with the goal of applying the sex and gender-specific knowledge into clinical practice to improve patient outcomes” (p181).^1^  As a tool for detecting and correcting the history of ignoring differences between the sexes due to practices such as excluding women from medical trials due to the potential for pregnancy, Song et al. (2016) created a SGSH searchable database which identified the subset of Medline-indexed articles likely to contain SGSH data using a combination of specific MeSH. Individual searches could then be applied to identify SGSH articles on specific diseases such as diabetes.^1^  By mapping the high-dimensional space representing all MeSH to a 2-d surface, the SOM allows for the simultaneous application of this technique to all medical knowledge simultaneously. It is possible to represent the degree of importance of subsets of MeSH, such as those used to generate the SGSH database above, across all nodes of a SOM by changing the weights of all other MeSH to 0, and representing the results in a density map (see Supplementary Figure 3).  Supplementary Figure 3 | | |
| --- | --- | --- |
| a)  Textbook SOM projection  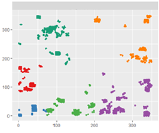 | b)  SGSH density map  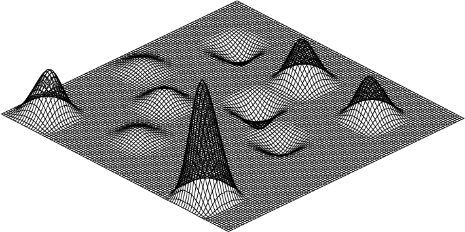 | c)  Density x SOM projection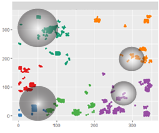 |
| *Note: Supplementary Figures 3a) and 3c) are taken from the main analysis of the paper, but the SGSH density map is purely illustrative; this analysis has not been done.* | | |
| Supplementary Figure 3 shows how the SOM makes it possible to superimpose the SGSH density map (b) representing the prominence of research featuring SGSH data across all the articles indexed by the Medline database, on the projection of the subset of knowledge contained within the KSCTP textbook (a). By considering only the peaks in the density map, which indicate the areas of knowledge with the most SGSH data, it becomes possible to identify the parts of the KSCTP addressed in the textbook which could start to consider differentiating sex and gender as part of the curriculum (c).  This method of identifying existing information about which curriculum developers were previously unaware is potentially useful for reducing implicit biases caused by a lack of knowledge. At a systemic level, superimposing the troughs of the SGSH density map on the SOM could also identify the areas of medical (or specifically psychiatric) knowledge with the least SGSH data. This would be expected for sexually dimorphic categories of disease, like prostate cancer or ovarian cancer, but it might indicate gaps in knowledge for diseases strongly affecting both sexes, such as heart disease.   1. Song MM, Simonsen CK, Wilson JD, Jenkins MR. Development of a PubMed Based Search Tool for Identifying Sex and Gender Specific Health Literature. J Women’s Health. 2016;25(2):181-187 | | |
